# Can self-testing be enhanced to hasten safe return of healthcare workers in pandemics? Random order, open label trial using two manufacturers’ SARS-CoV-2 lateral flow devices concurrently and nested viral culture study

**DOI:** 10.1186/s12879-024-10155-z

**Published:** 2024-11-11

**Authors:** Xingna Zhang, Christopher P. Cheyne, Christopher Jones, Michael Humann, Gary Leeming, Claire Smith, David M. Hughes, Girvan Burnside, Susanna Dodd, Rebekah Penrice-Randal, Xiaofeng Dong, Malcolm G. Semple, Tim Neal, Sarah Tunkel, Tom Fowler, Lance Turtle, Marta García-Fiñana, Iain E. Buchan

**Affiliations:** 1 Department of Public Health, Policy & Systems, University of Liverpool, Liverpool, UK; 2 Department of Health Data Science, University of Liverpool, Liverpool, UK; 3 Civic Health Innovation Labs (CHIL), University of Liverpool, Liverpool, UK; 4 Department of Infection Biology & Microbiomes, University of Liverpool, Liverpool, UK; 5 Department of Clinical Infection, Microbiology & Immunology, University of Liverpool, Liverpool, UK; 6 Centre for Educational Development & Support, University of Liverpool, Liverpool, UK; 7 Liverpool University Hospitals Foundation Trust, Liverpool, UK; 8 UK Health Security Agency, London, UK; 9 William Harvey Research Institute, Queen Mary University of London, London, UK; 10 Public Health Wales, Cardiff, UK

**Keywords:** Covid-19, SARS-CoV-2, Lateral flow test, Healthcare worker

## Abstract

**Background:**

Covid-19 healthcare worker testing, isolation and quarantine policies had to balance risks to patients from the virus and from staff absence. The emergence of the Omicron variant led to dangerous levels of key-worker absence globally.

We evaluated whether using two manufacturers’ lateral flow tests (LFTs) concurrently improved SARS-CoV-2 Omicron detection significantly and was acceptable to hospital staff. In a nested study, to understand risks of return to work after a 5-day isolation/quarantine period, we examined virus culture 5–7 days after positive test or significant exposure.

**Methods:**

Fully-vaccinated Liverpool (UK) University Hospitals staff participated (February-May 2022) in a random-order, open-label trial testing whether dual LFTs improved SARS-CoV-2 detection, and whether dual swabbing was acceptable to users. Participants used nose-throat swab Innova and nose-only swab Orient Gene LFTs in daily randomised order for 10 days. A user-experience questionnaire was administered on exit. Selected participants gave swabs for viral culture on days 5–7 after symptom onset or first positive test. Cultures were considered positive if cytopathic effect was apparent or SARS-CoV-2 N gene sub-genomic RNA was detected.

**Results:**

Two hundred and twenty-six individuals reported 1466 pairs of LFT results. Tests disagreed in 127 cases (8.7%). Orient Gene was more likely (78 cf. 49; OR: 2.1, 1.1–4.1; *P* = 0.03) to be positive. If Innova was swabbed second, it was less likely to agree with a positive Orient Gene result (OR: 2.7, 1.3–5.2; *P* = 0.005); swabbing first with Innova made no significant difference (OR: 1.1, 0.5–2.3; *P* = 0.85). Orient Gene positive Innova negative result-pairs became more frequent over time (OR: 1.2, 1.1–1.3; *P* < 0.001).

Of individuals completing the exit questionnaire, 90.7% reported dual swabbing was easy, 57.1% said it was no barrier to their daily routine and 65.6% preferred dual testing. Respondents had more confidence in dual versus single test results.

Viral cultures from days 5–7 were positive for 6/31 (19.4%, 7.5%-37.5%) and indeterminate for 11/31 (35.5%, 19.2%-54.6%) LFT-positive participants, indicating they were likely still infectious.

**Conclusions:**

Dual brand testing increased LFT detection of SARS-CoV-2 antigen by a small but meaningful margin and was acceptable to hospital workers. Viral cultures demonstrated that policies recommending safe return to work ~ 5 days after Omicron infection/exposure were flawed. Key-workers should be prepared for dynamic self-testing protocols in future pandemics.

**Trial registration:**

https://www.isrctn.com/ISRCTN47058442 (26 January 2022).

**Supplementary Information:**

The online version contains supplementary material available at 10.1186/s12879-024-10155-z.

## Background

**Fig. 1:**
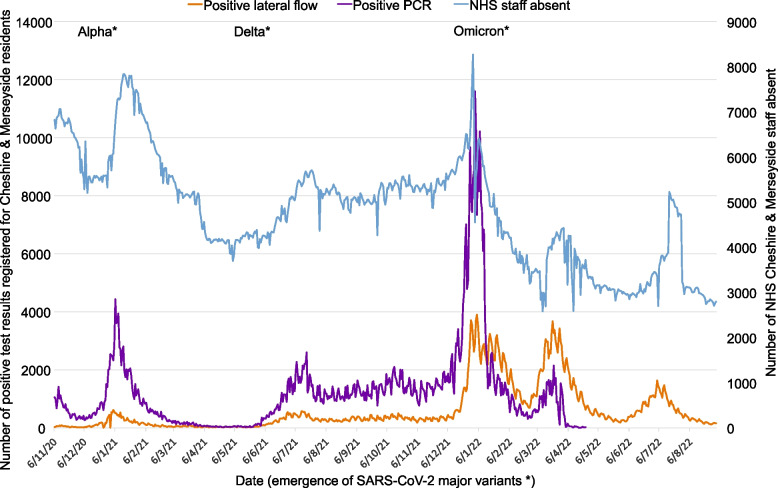
Numbers of NHS staff absent, and numbers of positive PCR and lateral flow test results reported for residents of Cheshire & Merseyside, UK from the start of introduction of lateral flow community testing to the end of the study period

The Covid-19 pandemic stretched health systems worldwide [1, 2]. Healthcare workers suffered high rates of infection and mortality [3–5], and policymakers faced dilemmas in balancing risks. In late 2021, as Omicron hit the UK, hospitalised patients faced risks from having too few staff to care for them (Fig. 1), which potentially posed a greater threat to patient safety and care delivery than Covid-19 itself [6–10]. Omicron’s increased transmissibility and immune evasion demanded a rethink of Covid-19 policies for healthcare workers and the public [10–13].

Pre-Omicron, UK healthcare workers required a negative PCR 10 days from exposure to return from quarantine [14, 15]. Waiting (typically 48-h) for PCR results delayed return to work [15], and PCR capacity affected care-service continuity [16, 17]. By December 2021, it was evident that SARS-CoV-2 lateral flow tests (LFTs) were reasonable and affordable indicators of infectiousness [18–20]. LFTs from some manufacturers used nose-only swabbing, others nose-throat swabbing, with nose-only testing assumed to have better compliance. Policymakers were concerned that nose-only swabbing might delay detection of Omicron, which was reportedly shed from the throat ahead of the nose [21] – a concern not addressed by national testing quality assurance programmes [22, 23].

In December 2021 and January 2022, NHS staff testing policies changed to address staff shortages. Based on mathematical modelling, NHS workers were permitted to return from isolation or quarantine: after two consecutive days of negative LFTs beyond 5 days since exposure or first positive test; or if still testing positive, 10 days from symptom onset or first positive test, provided they felt well enough [14, 15, 24]. This guidance was updated on 7th January 2022 to advise local risk assessments for those testing positive on days 10–14 [25].

The modelling of serial negative LFT results to inform return to work was performed by the Scientific Pandemic Influenza Group on Modelling (SPI-M) [26] and UK Health Security Agency (UKHSA) [27] alongside unpublished viral culture studies for the New and Emerging Respiratory Virus Threats Advisory Group (NERVTAG).

This study was commissioned by the UK Covid-19 Testing Initiatives Evaluation Board (TIEB) to extend its testing quality assurance programme. We investigated whether SARS-CoV-2 antigen detection in daily self-testing was improved by using kits from two manufacturers concurrently; one requiring nose-only and one nose-throat swabbing [22]. Real-world testing sensitivity and NHS staff acceptability were the main outcomes. A nested virus culture study assessed the infectiousness of individuals still testing positive after day-5 since symptom onset or first positive test, as the US policy was to return to work after day-5 without testing. Data from this study informed UK policies via TIEB [28].

## Methods

### Aim

We aimed to evaluate effectiveness and acceptability of dual versus single brand SARS-CoV-2 antigen lateral flow self-testing among hospital workers, and to determine whether culturable SARS-CoV-2 Omicron was present 5–7 days after a positive test or significant exposure.

### Trial design

An open-label, randomised-order trial of using two LFT brands concurrently in daily self-testing with the ‘Test-to-Release’ [29], or Daily Contact Testing design [30–33].

### Setting

Participants comprised fully vaccinated [34] NHS workers using Covid-19 staff-testing facilities for contacts or cases at Liverpool University Hospitals NHS Foundation Trust, UK between 7th February and 8th May 2022. Participants entered the study by booking a test routinely on-line where they received study information and consented to participate. Participants were recruited via one of three pathways (Appendix 1) depending on their status at baseline: i) test-negative close contact (reflecting routine daily testing as an alternative to quarantine for an uninfected staff member notified as a close contact of a known case); ii) test-positive symptomatic (reflecting routine isolation of a staff member presenting for testing due to symptoms and testing positive); or iii) test-positive asymptomatic (reflecting routine isolation of a staff member without symptoms testing positive, including via routine daily contact testing in quarantine and additional tests from this study). Data were collected via on-line questionnaires and NHS record linkage.

### Intervention

The study used two LFT brands widely available via NHS Test & Trace in February 2022: the nose-only swab Orient Gene and nose-throat swab Innova (Xiamen Biotime Biotechnology) kits. These have similar performance curves versus viral load when compared to PCR results [23].

Participants were asked to take two LFTs daily for 10 days, and on day-1 and day-5 to return swabs for quantitative PCR. Test order was detailed on an information sheet (Appendix 2), with daily LFTs in random order (Innova or Orient Gene first) and PCR on day-1 and day-5. Participants uploaded LFT results via NHS Test & Trace systems – with automated image reading available via the national standard app to ease consistent reporting – participants could override the automated reading: no data were available on overrides but previous studies suggest these were rare [35].

A nested study considered culture of viable virus at days 5–7 from first positive test.

### Outcomes

The primary outcome was the discordance of results from concurrent LFTs. Secondary outcomes were participant compliance, and self-reported experience of dual versus single testing from the exit survey detailed in Appendix 3.

### Sample size

**Fig. 2:**
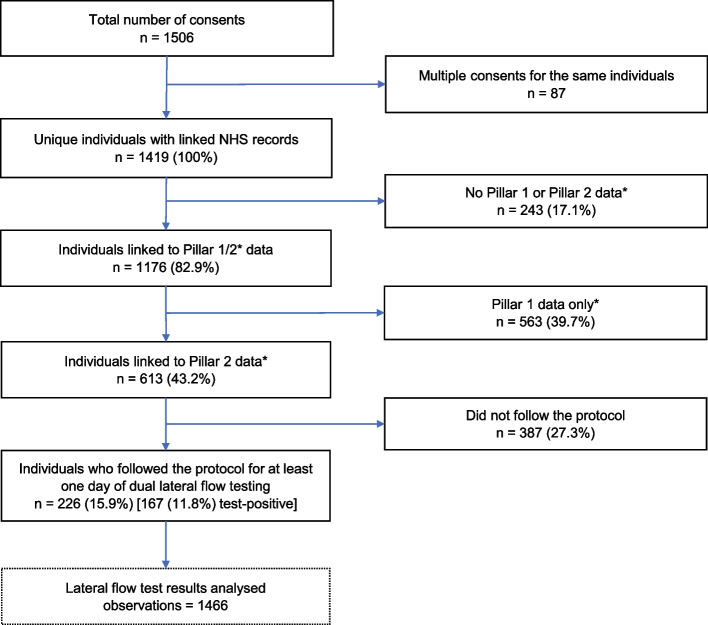
Flow of participants from consent to data analysed. *Pillar 1 comprised PCR tests processed in hospital laboratories. Pillar 2 comprised PCR tests processed in national NHS Test & Trace laboratories and lateral flow test results reported by individuals self-testing and putting results into the national NHS website or app

Calculations (see Appendix 4) assumed 18% drop-out and 10% test-positivity. The proportion of consented individuals not returning data was higher than expected (Fig. 2), and test-positivity was > 10%. Power to detect a difference between dual and single testing was the main target and the number of participants testing positive (*n* = 167) was similar to the number required (*n* = 164). It was later reported that SARS-CoV-2 LFTs were more sensitive to Omicron than prior variants, with Orient Gene more sensitive than Innova [23].

### Viral culture and sequencing to determine lineage

Appendix 5 details viral culture, RNA-extraction, sequencing and bioinformatics methods used to infer the presence of replicable SARS-CoV-2 lineages from swab samples. In brief, Calu3 cells, cultured at 10^5 cells/well in 24 well plates, were inoculated for viral culture, incubated and checked for cytopathic effect (CPE) after three days. If CPE was visible supernatants were sampled for RNA extraction. If no CPE was visible a 2nd passage was performed before supernatant sampling. RNA was extracted from supernatants and used for amplicon sequencing by MinION, using a published method. Fastq reads were analysed using the ARTIC [36] bioinformatic pipeline and lineages were called with Pangolin [37]. LeTRS was used to assess the presence of N gene sub genomic RNA (sgRNA) [38], indicative of active viral transcription.

### Statistical methods

Discordance of result-pairs from two LFT brands was analysed with McNemar’s test, including Yang’s adjustment and logistic mixed-effects models to account for test-clustering within individuals over time and in study-day groups [39]. Trends over time in discordance were analysed with a logistic mixed-effects model addressing clustering within individuals with study-day groups disaggregated.

Comparison of users’ confidence in single versus dual testing from questionnaire ordinal score data used a Wilcoxon signed ranks test and exact confidence interval as score distributions were skewed. Confidence intervals for binomial proportions used the Clopper-Pearson method, and for logistic mixed-effects models the Wald method. Analyses were performed using R version 4.3.1. Results were verified independently by two statisticians. Results are presented as main effect with 95% confidence interval.

### Patient and public involvement

UKHSA’s Research Ethics & Governance of Public Health Practice Group (REGG), including lay members, along with TIEB, fed back on drafts of the study protocol as part of the approvals process. Additional public involvement over data governance was provided by Liverpool City Region Civic Data Cooperative.

## Results

### Main outcomes

**Fig. 3:**
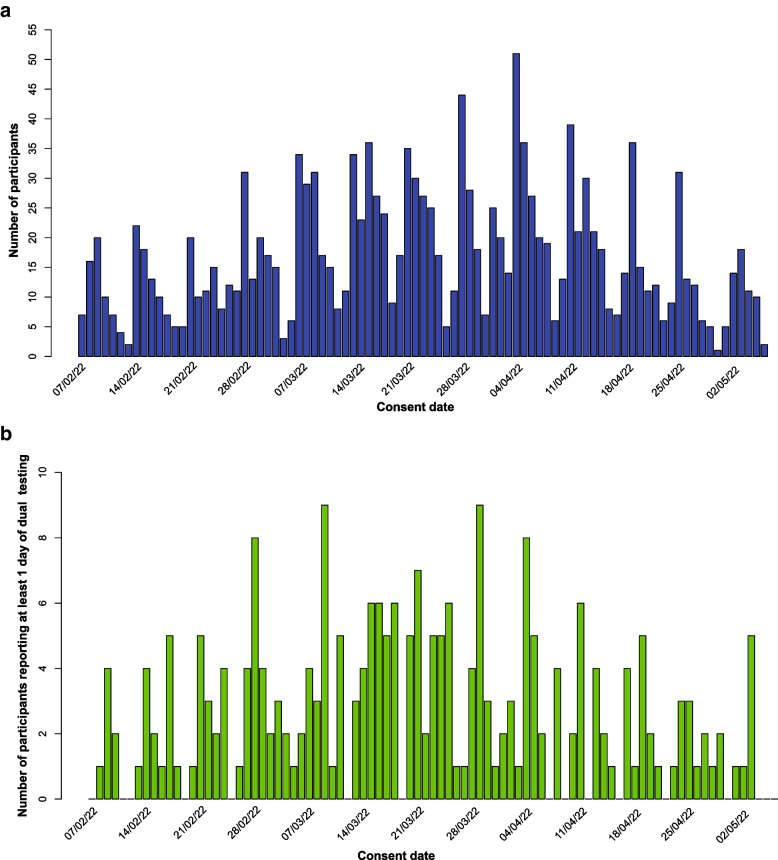
A Number of participants consenting to take part in the study by consent date. **B** Recruitment pattern over time of 226 participants who completed at least one day of dual lateral flow testing over the study period

Two hundred and twenty-six participants reported at least one day of dual LFT results between study day-1 and day-10, giving 1466 pairs of tests. Figure 2 shows the flow of participants from consent to analysis, Fig. 3 the recruitment patterns over time.

The study population comprised 226 participants reporting 1466 pairs of LFT results. 167 (73.9%) of the 226 participants were classified as cases at any point during the study – a) at baseline if they had a positive PCR from the staff testing centre where they consented to participate or reported dual LFT positive results on day 1; and b) if they reported dual LFT positive results on any of days 2–10. PCR results were available for 190 (84.1%) at recruitment/consent and inferred from day 1 LFT results for a further 21 leading to a total of 211 (93.4%) having a definitive infection status at baseline. 46 (20.4%) reported dual LFT results for the full 10 days, where others tended to stop reporting results after turning from positive to negative. Appendix 6 gives further accrual details.

A total of 125 individuals had a positive PCR test at recruitment/consent. In this group, the first reported LFT result pairs were both positive for 116 (92.8%), both negative for 3 (2.4%), Innova positive only for 3 (2.4%) and Orient Gene positive only for 3 (2.4%) – a discrepancy rate (~ detection uplift from dual versus single testing) of 6/125 (4.8%, 1.8%-10.2%). On day 2, 99 were positive on both LFTs (90%), 5 were negative on both (4.5%), 5 were Orient Gene positive only (4.5%), and 1 was Innova positive only (1%) – a discrepancy rate of 6/110 (5.5%, 2.0%-11.5%). Considering two days of consecutive test results, only 2 (1.6%) individuals would have been LFT negative and PCR positive within 48 h.

Of the 1466 pairs of LFT results reported, 127 (8.7%) were discordant (gave different results). For those participants who tested positive at any time point (*n* = 167, 73.9%), on those days where they tested positive using dual LFT testing (*n* = 866), the number of pairs where the first recorded test was negative was 46 (5.3%).

Overall, Orient Gene had double the odds of being positive compared to Innova when the two tests disagreed (Table 1). 156 (93.4%; 88.5%-96.7%) suspected infections were detected with Orient Gene compared to 163 (97.6%; 94.0%-99.3%) with Innova. Out of the test-positive cohort of 167, 59 (35.3%) had at least one subsequent period of 2 or more consecutive days of dual negative LFTs. Of these, none had a pair of positive LFT results afterwards.

**Table 1:** Lateral flow test results by brand

		Innova		
		Negative	Positive	Total
Orient Gene	Negative	596 (40.7%)	49 (3.3%)	645
	Positive	78 (5.3%)	743 (50.7%)	821
	TOTAL	674	792	1466 (100%)

Test pairs with any equivocal result were excluded. McNemar (Yang-adjusted) Chi^2^ = 4.636, *P* = 0.03, logistic mixed-effects model for discordant tests odds ratio (OR) = 2.1(1.1–4.1), *P* = 0.03

When Orient Gene was the first test (Table 2), Orient Gene positive Innova negative was a more likely discordant result than Innova positive Orient Gene negative (OR = 2.7, 1.3–5.2; *P* = 0.005). No significant difference was observed when Innova was the first test (OR = 1.1, 0.5–2.3; *P* = 0.85, Table 3). Direct comparison of discordant test pairs shows the odds of an Orient Gene positive with Innova negative discordance was 4.5 times higher when Orient Gene was first versus Innova first (OR = 4.5, 1.1–18.1; *P* = 0.04).

**Table 2:** Lateral flow test results when Orient Gene was recorded first

		Innova		
		Negative	Positive	Total
Orient Gene	Negative	307 (40.3%)	18 (2.4%)	325
	Positive	47 (6.2%)	390 (51.2%)	437
	Total	354	408	762 (100%)

Test pairs with any equivocal result were excluded. McNemar (Yang-adjusted) Chi^2^ = 11.668, *P* = 0.001, logistic mixed-effects model for discordant tests OR = 2.7(1.3–5.2), *P* = 0.005

**Table 3:** Lateral flow test results when Innova was recorded first

		Innova		
		Negative	Positive	Total
Orient Gene	Negative	289 (41.1%)	31 (4.4%)	320
	Positive	31 (4.4%)	353 (50.1%)	384
	TOTAL	320	384	704 (100%)

Test pairs with any equivocal result were excluded. McNemar (Yang-adjusted) Chi^2^ < 0.001, *P* > 0.99, logistic mixed-effects model for discordant tests OR = 1.1(0.5–2.3), *P* = 0.85

**Fig. 4:**
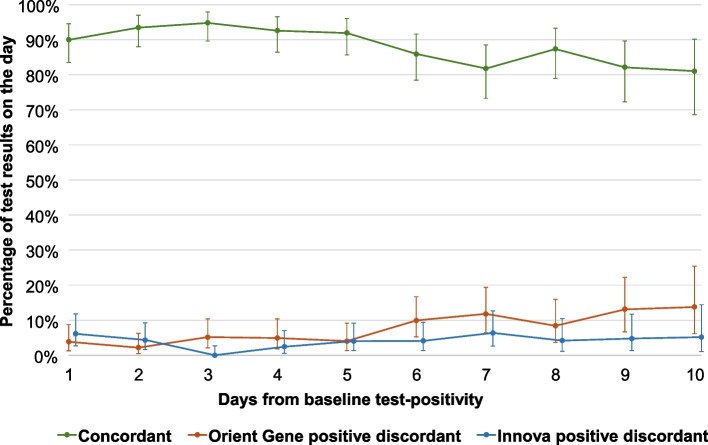
Percentage of concordant and discordant lateral flow test result pairs by brand and days from baseline for those testing positive

Of the 167 participants who tested PCR or LFT positive at study entry or became LFT test-positive during the study (Table 4), the proportion of Orient Gene positive discordant tests increased significantly over time (OR: 1.2, 1.1–1.3; *P* < 0.001), and not significantly for Innova (OR: 1.1, 0.97–1.2; *P* = 0.15). Direct comparison of the two discordant groups using a logistic mixed effects model did not show statistical significance (OR: 1.2, 0.99–1.6; *P* = 0.07), however, small numbers of discordant groups (Fig. 4) may have limited the power to resolve this effect. For participants who never tested positive, there was very little discordance between test results (Appendix 6: Table A6.6).

**Table 4:** Dual lateral flow test (LFT) results by brand and days from baseline (study entry or first positive test) for individuals who tested positive

	**Day from baseline test-positivity**									
Dual LFT results	**1**	**2**	**3**	**4**	**5**	**6**	**7**	**8**	**9**	**10**
Concordant	117 (90.0%)	129 (93.5%)	128 (94.8%)	113 (92.6%)	114 (91.9%)	104 (86.0%)	90 (81.8%)	83 (87.4%)	69 (82.1%)	47 (81.0%)
*Both positive*	*114* *(87.7%)*	*122* *(88.4%)*	*123* *(91.1%)*	*103* *(84.4%)*	*94* *(75.8%)*	*73* *(60.3%)*	*52* *(47.3%)*	*37* *(38.9%)*	*20* *(23.8%)*	*5* *(8.6%)*
*Both negative*	*3* *(2.3%)*	*7* *(5.1%)*	*5* *(3.7%)*	*10* *(8.2%)*	*20* *(16.1%)*	*31* *(25.6%)*	*38* *(34.5%)*	*46* *(48.4%)*	*49* *(58.3%)*	*42* *(72.4%)*
Discordant	13 (10.0%)	9 (6.5%)	7 (5.2%)	9 (7.4%)	10 (8.1%)	17 (14.0%)	20 (18.2%)	12 (12.6%)	15 (17.9%)	11 (19.0%)
*Orient Gene positive*	*5* *(3.8%)*	*3* *(2.2%)*	*7* *(5.2%)*	*6* *(4.9%)*	*5* *(4.0%)*	*12* *(9.9%)*	*13* *(11.8%)*	*8* *(8.4%)*	*11* *(13.1%)*	*8* *(13.8%)*
*Innova positive*	*8* *(6.2%)*	*6* *(4.3%)*	*0* *(0.0%)*	*3* *(2.5%)*	*5* *(4.0%)*	*5* *(4.1%)*	*7* *(6.4%)*	*4* *(4.2%)*	*4* *(4.8%)*	*3* *(5.2%)*
Total	130	138	135	122	124	121	110	95	84	58

Table includes only those participants who tested PCR or LFT positive at study entry or became LFT test-positive throughout the study and counts dual test results they reported on any day. Any LFT pair with any equivocal result was excluded, along with any dual tests prior to the first recorded positive test for each participant

### Viral culture analysis

Viral cultures were analysed for 41 participants (see Appendix 5 for details): 31 continuing LFT positive and 10 reverting negative on days 5–7. 6/31 (19.4%, 7.5%-37.5%) of the continued LFT positives were culture positive, 9/31 (29.0%, 14.2%-48.1%) were indeterminate by cytopathic effect (CPE), none of these had N-sgRNA detected by sequencing. Two additional cultures with no CPE had N-sgRNA detected, both at low level. 2/10 (20.0%, 2.5%-55.6%) of the reverting LFT negatives were indeterminate by CPE with N-sgRNA not detected by sequencing. Two cultures without CPE from LFT negative individuals had N-sgRNA detected at low levels. PCR was carried out on the original swab aliquot: all came back SARS-CoV-2 positive, with S Gene target present in 36 (likely Omicron BA.2) and absent in 5 (likely Omicron BA.1).

### Exit questionnaire survey

Three hundred and eleven participants responded to the exit survey between 10th February and 20th July 2022 (details in Appendix 5). 229 (73.7%) identified as a woman, 77 (24.7%) as a man, 0 as non-binary and 5 (1.58%) preferred not to say. Professions were: 57 (18.5%) doctors; 84 (27.2%) nurses; 78 (25.0%) allied health professionals; 25 (8.2%) clinical support staff; 44 (14.1%) administration/clerical staff; 22 (7.07%) other staff. When asked “how easy was the swabbing process” 154 (49.6%) selected “very easy”, 128 (41.2%) “easy”, 26 (8.4%) “neither easy nor difficult”, 3 (0.9%) “difficult”, and none “very difficult”. When asked “How much of a barrier is having to take a throat as well as a nose swab for your daily rapid test?” 178 (57.1%) selected “not at all”, 80 (25.6%) selected “slight barrier”, 38 (12.3%) selected “somewhat of a barrier”, 12 (3.94%) selected “moderate barrier”, and 3 (1.0%) selected “extreme barrier”. When asked “Could you fit taking two rapid tests into your daily routine within an hour of leaving for work?” 90 (28.9%) respondents selected “definitely”, 58 (18.6%) selected “very probably”, 110 (35.3%) selected “probably”, 44 (14.2%) selected “probably not”, and 9 (2.9%) selected “definitely not”. When asked “If you were asked to continue to do two tests daily instead of one, would you?” 204 (65.6%) selected “yes”, 57 (18.2%) selected “no”, and 50 (16.2%) selected “no preference”. When asked “which mode of testing are you most confident about” and given a rating scale from 1 = “no confidence” to 10 = “full confidence” for “single test” and “double test” the median scores were 8 for single test and 9 for double test – a median difference of 1 (0.5 to 1; *P* < 0.001) – a small but psychologically significant difference.

## Discussion

### Main findings

We found that practical combination of LFT brands with different swab types enhanced antigen detection differently at different time points during infection. Combining Orient Gene and Innova LFTs improved SARS-CoV-2 (Omicron BA.1/2) antigen detection by a small amount [4.8% (1.8%-10.2%)] compared to a single test. This marginal effect was relevant for managing safe return to work amid potentially dangerous staff shortages.

Orient Gene was more likely to be the sole positive test – with Orient Gene positive Innova negative results becoming more frequent over successive days. If Innova was swabbed second it was less likely to agree with a positive Orient Gene result; swabbing first with Innova made no significant difference.

Dual testing was largely acceptable to hospital staff, with most reporting dual swabbing manageable. Almost two-thirds preferred to continue dual testing if required and had slightly more confidence in dual versus single testing results. Over half said dual swabbing was no barrier to daily routines. However, the two LFT kits required different swabbing methods and around 40% of staff reported that swabbing both nose and throat was at least a slight barrier.

Almost a fifth of individuals with positive LFTs at days 5–7 had positive culture, indicating they were likely still infectious. We did not identify any convincingly culture positive individuals who had a negative LFT by this time, though we could still detect viral RNA sequences.

### Comparison with other studies

Evidence on end-to-end risk mitigation using LFTs in the Covid-19 pandemic is limited, with a few small serial testing and viral culture studies nested within studies comparing LFT and PCR performance [23, 40, 41]. One study found minimal improved sensitivity by using the same manufacturer's LFT in rapid succession [42]. Ours is the only real-world trial, to our knowledge, of combining LFTs to enhance risk-mitigation in balancing risks from Covid-19 versus healthcare staff shortages. The shorter incubation period of Omicron compared with earlier variants challenged previous risk-mitigations [43, 44] and there were concerns over later nasal shedding invalidating nose-only swab LFTs [21], which we showed were mitigated by dual testing.

Viral culture studies with early variants found that people infected with SARS-CoV-2 became infectious 1–2 days before the onset of symptoms and remained infectious until 7 days later [43, 45]. Our data showed a substantial proportion of individuals were potentially infectious beyond this point. Most work on this topic has placed too much emphasis on the median duration of infection at the expense of considering variability and its impact on fixed time-period policies for return from isolation or quarantine.

### Strengths and limitations of this study

This is the first study, to our knowledge, to compare dual with single brand LFT self-testing of healthcare workers in managing the risks from Covid-19 versus under-staffed care due to high numbers isolating or quarantined. It was performed when the UK was under pressure from Omicron variants in early 2022. It was thus a realistic test of enhanced risk-mitigation and comprehensively considered LFT sensitivity, participant experience, and security (using viral cultures) of prompt return to work. The 10-day observation period allowed assessment of the contemporary Covid-19 policies for healthcare worker release from isolation and quarantine. The results give insights into the combined performance of two brands of LFT, which cannot be inferred from the usual comparison with concurrent PCR tests [23, 46].

Our study had limitations: slow research approvals meant the peak of the initial Omicron epidemic was missed. Staff burnout and low morale slowed recruitment and prolonged the study duration. National policies for NHS staff testing changed several times during the study [13, 47], and public access to LFTs was reduced on 1st April 2022 [47], potentially also impeding participant uptake. These barriers reflect the real-world challenges of evaluating new risk-mitigations two years into a pandemic and under winter pressures.

Only 16% of consented participants followed the protocol for at least one day – this likely limits the representativeness of the survey results, which also don’t reflect the hospital staff who didn’t wish to participate in the study. Finally, viral culture interpretation was limited – parallel PCR swabbing and sequencing would have given more confidence that SARS-CoV-2 was present and not another virus.

### Policy implications

Throughout the Covid-19 pandemic, policies for testing healthcare workers changed many times [10, 13, 16, 48]. In the UK, LFT self-testing became part of life. However, with the rise of Omicron policymakers feared that nose-only LFT swabbing may miss numerous infections. Our study allayed these fears, showing reasonable concordance of the widely available Orient Gene nose-only and Innova nose-throat swab LFTs. Policymakers were therefore right to use all available stocks of LFTs, including those with nose-only swabs, to mitigate elevated risks.

Despite pandemic pressures, study participants reported largely positive experiences of using two LFTs instead of one for daily self-testing. The resulting improvement in detection was small yet meaningful in a universal health system coordinating risk-mitigations system-wide.

Internationally, there was pressure to balance risks from Covid-19 with those from mass absence of key workers. Our viral culture study shows that fixed isolation time policies, such as the US advice to return to work 5 days after testing positive, were flawed [49]. The UK’s ‘test-to-release from isolation’ (after two days of negative LFT results) policy, formed in December 2021, was reasonable given staffing pressures at the time.

The speed, convenience, and socialisation of LFT self-testing in the UK allowed enhanced Covid-19 risk-mitigation under pressure from Omicron. A better UK response would have extended testing quality assurance from public health agencies to the NHS. Ideally, more serial samples of daily antigen, nucleic acid and culturable virus testing would have informed policy modelling. Future pandemic preparations globally should consider closer surveillance of serial self-testing to inform evolving risk-mitigations. In addition, dynamic economic evaluations should be built into the monitoring of large-scale pandemic testing programmes.

## Conclusion

Policymakers’ fears that nose-only LFT swabbing may miss a substantial proportion of Omicron BA.1/2 infections were allayed by our study of NHS workers in the UK between February and June 2022. Combining the widely available Innova nose-throat and Orient Gene nose-only LFT kits increased Omicron detection and was acceptable to participating hospital staff self-testing in isolation or quarantine. This improvement was small yet meaningful in a universal health system coordinating risk-mitigations system-wide. The US policy of return to work 5 days after testing positive was shown by our viral culture results to be flawed. The speed, convenience, and public socialisation of LFT self-testing in the UK allowed enhanced Covid-19 risk-mitigation during the pressures caused by the Omicron variant. A better UK response would have extended testing quality assurance from public health agencies to the NHS. Future pandemic preparedness may be enhanced by continuous surveillance of serial self-testing, considering end-to-end risk mitigation as well as technology performance.

## Supplementary Information

Supplementary Material 1.

## Data Availability

The study required person identifiable data and the main analyses were conducted on a de-identified extract. The fully anonymised data for reproducing the results are available from https://github.com/iain-buchan/cipha/blob/master/SMART_RR_Anonymised_Data.zip. The study protocol can be downloaded from https://github.com/iain-buchan/cipha/blob/master/SMART_Release_Return.pdf and statistical analysis plan from https://github.com/iain-buchan/cipha/blob/master/SMART_RR_SAP.pdf.

## Abbreviations

Covid-19: Coronavirus disease 2019
SARS-CoV-2: Severe acute respiratory syndrome coronavirus 2
LFT: Lateral flow test
CPE: Cytopathic effect
NHS: UK National Health Service
TIEB: UK Covid-19 Testing Initiatives Evaluation Board
UKHSA: UK Health Security Agency
NERVTAG: New and Emerging Respiratory Virus Threats Advisory Group
CDC: US Centres for Disease Control and Prevention
