# Supplementary material for: Can self-testing be enhanced to hasten safe return of healthcare workers in pandemics? Random order, open label trial using two manufacturers’ SARS-CoV-2 lateral flow devices concurrently and nested viral culture study
Source: BMC Infect Dis. 2024 Nov 11;24:1276. doi: 10.1186/s12879-024-10155-z (PMC11555827; doi:10.1186/s12879-024-10155-z)
Supplement: Supplementary file 1 — Supplementary Material 1. [file 12879_2024_10155_MOESM1_ESM.docx]

**Appendix 1: Participant Pathways**

The full study protocol is available from:
<https://github.com/iain-buchan/cipha/blob/master/SMART_Release_Return.pdf>

## **A. Uninfected contact participant pathway (illustrated in Figure A1.1)**

1. Household member of NHS worker was notified they were Covid positive, so their NHS contact started quarantine and notified their employer.
2. Employer had adopted SMART Release & Return testing schedule as their local standard policy and directed the staff member to a booking website for the scheme, which provided information sheet, consent process and directions to the unit/site.
3. Participant received a 10-day pack of daily dual LFTs and 2 PCR home test kits, and if they had not had a positive Covid test in the past 90 days they took a swab for quick turnaround (binary) PCR.
4. Participant received PCR negative result on day 0 and returned to work on day 1 with DCT.
5. Either Innova (nose/throat) or Orient Gene (nose only) LFTs were taken each morning (or pre-shift) before breakfast in randomised order for 10 days – an information sheet in the pack directed the participant day by day. Either LFT reporting positive was an overall positive result.
6. On day 1 the participant also took home a PCR swab (randomised order with the two LFTs) and returned it by post to Pillar 2 / other (ringfenced) Q-RT-PCR capacity, and the result was not used for any purpose other than research.
7. A second Q-RT-PCR swab was taken on day 5.
8. Exit questionnaire gathered participant experiences.


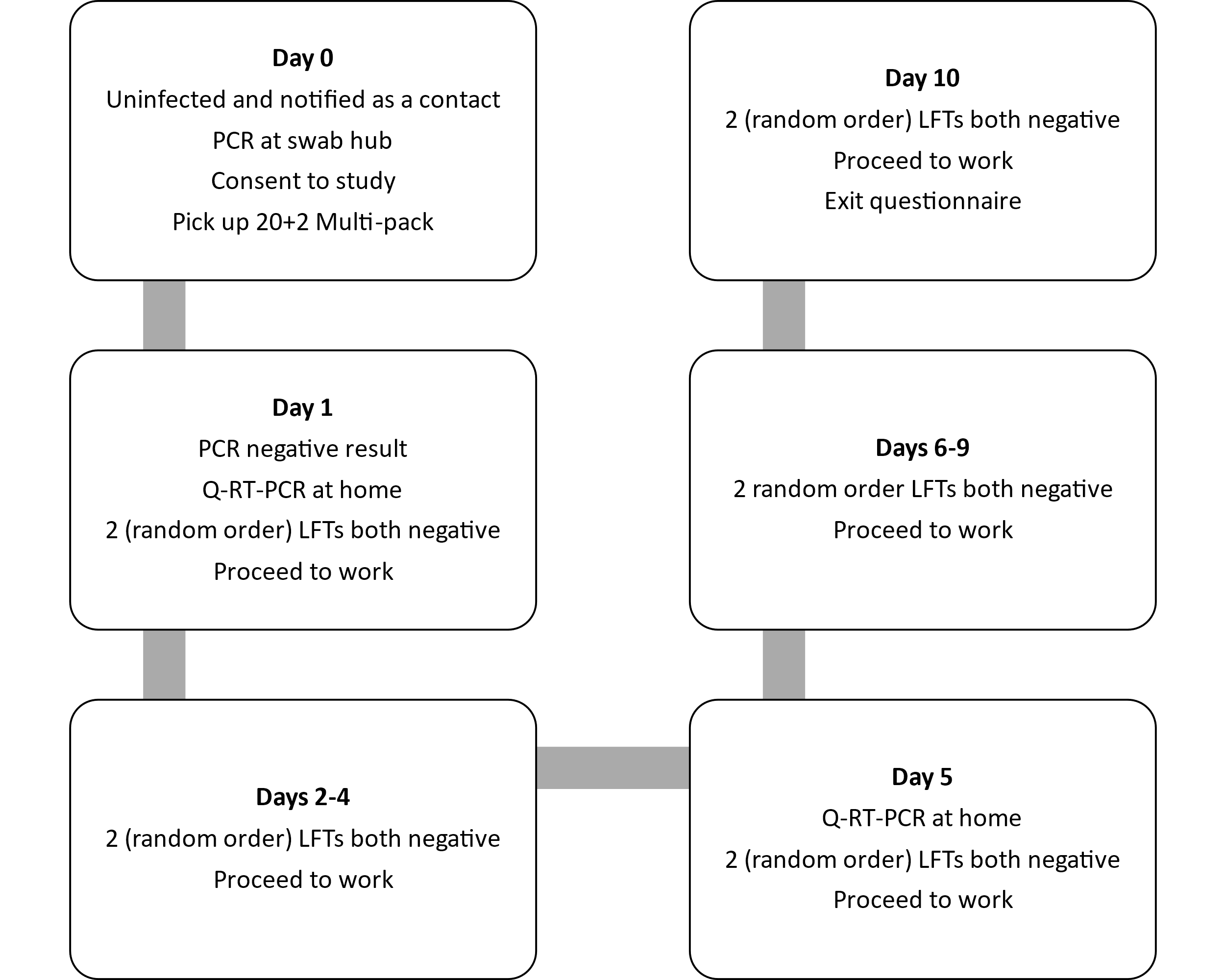


**Figure A1.1. Workflow for participant who tested negative throughout the study**

## **B. Asymptomatic infected contact participant pathway (illustrated in Figure A1.2)**

1. Household member of NHS worker was notified they were Covid positive, so their NHS contact started quarantine and notified their employer.
2. Employer had adopted SMART Release & Return testing schedule as their local standard policy, directed the staff member to a booking website for the scheme, and directed them to the standard testing/reception site.
3. Consented participant took quick turnaround (binary) PCR test to return to work from quarantine on DCT and received a 10-day pack of daily dual LFT + 2 PCR home test kits.
4. Participant received PCR positive result on day 0 and stayed at home.
5. Either Innova (nose/throat) or Orient Gene (nose only) LFTs were taken each morning before breakfast in randomised order – an information sheet in the pack directed the participant day-by-day.
6. On day 1 the participant also took home a PCR swab (randomised order with the two LFTs) and returned it by post to Pillar 2 / other (ringfenced) Q-RT-PCR capacity, and the result was not used for any purpose other than research.
7. Second Q-RT-PCR swab was taken on day 5. (Participant was selected to be in the viral culture sample of 30 cases – and their swab in viral transport medium was collected from their home).
8. If day 5 and 6 dual LFT results (4 tests) were negative the participant may return to work.
9. Daily dual LFT testing continued until day 10.
10. If still testing LFT positive at day 7 the participant was advised to call and arrange a RT-Q-PCR swab in viral transport medium for culture.
11. Exit questionnaire gathered participant experiences.


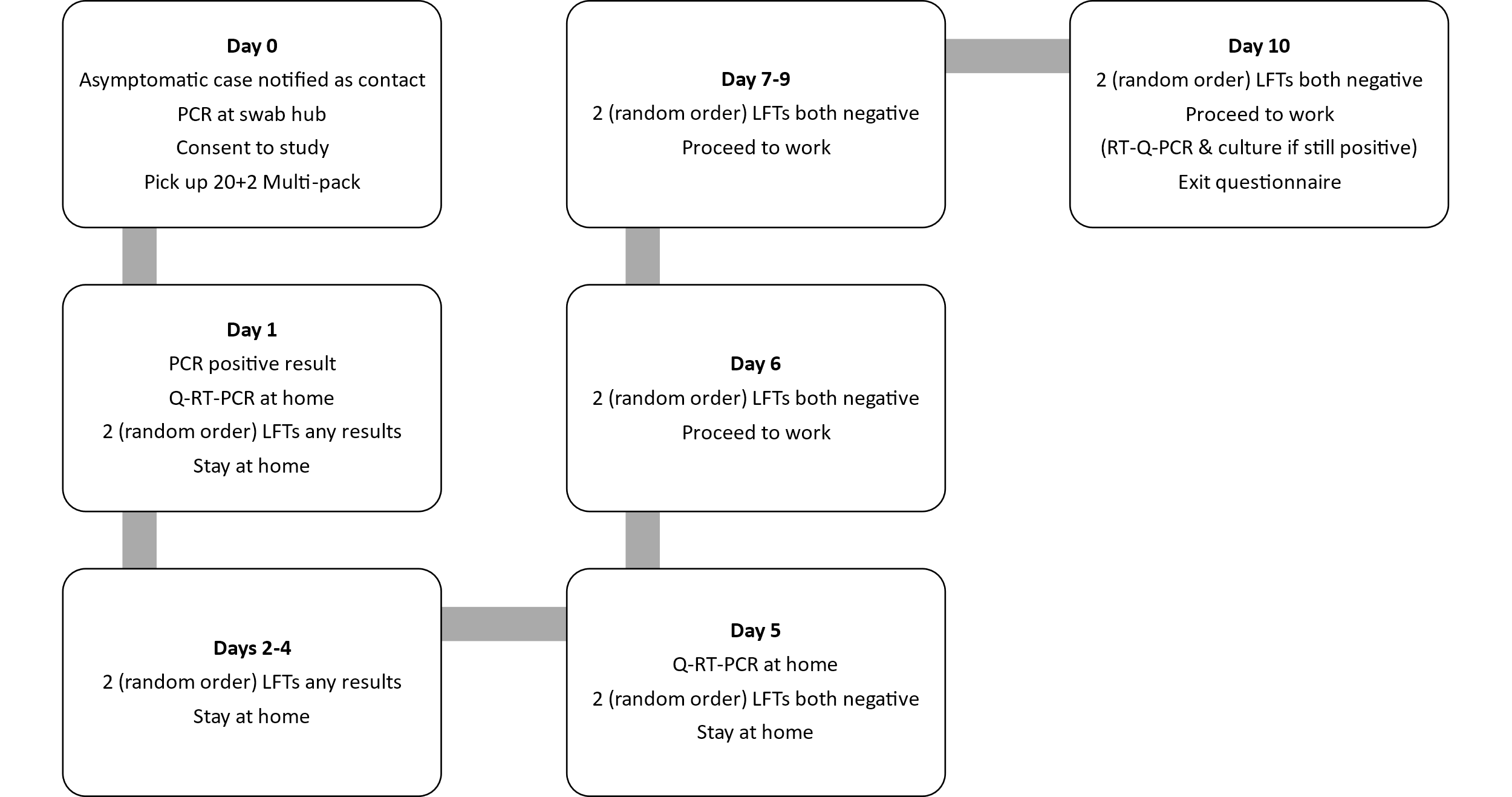


**Figure A1.2. Workflow for contact who tested positive in the beginning of the study**

## **C. New case referred to the study (illustrated in Figure A1.3)**

1. NHS worker was notified they were Covid positive and notified their employer.
2. Employer had adopted SMART Release & Return testing schedule as their local standard policy, directed the staff member to a booking website for the scheme, and directed them to the standard testing/reception site.
3. Consented participant received a 10-day pack of daily dual LFT + 2 PCR home test kits.
4. Innova (nose/throat) and Orient Gene (nose only) LFTs were taken each morning before breakfast in randomised order – an information sheet in the pack directed the participant day-by-day.
5. On day 1 the participant also took home a PCR swab (randomised order with the two LFTs) and returned it by post to Pillar 2 / other (ringfenced) Q-RT-PCR capacity, and the result was not used for any purpose other than research.
6. Second Q-RT-PCR swab was taken on day 5.
7. If Day 5 and 6 dual LFT results (4 tests) were negative the participant may return to work.
8. Daily dual LFT testing continued until day 10.
9. If still testing LFT positive at day 7 the participant was advised to call and arrange a RT-Q-PCR swab in viral transport medium for culture.
10. Exit questionnaire gathered participant experiences.


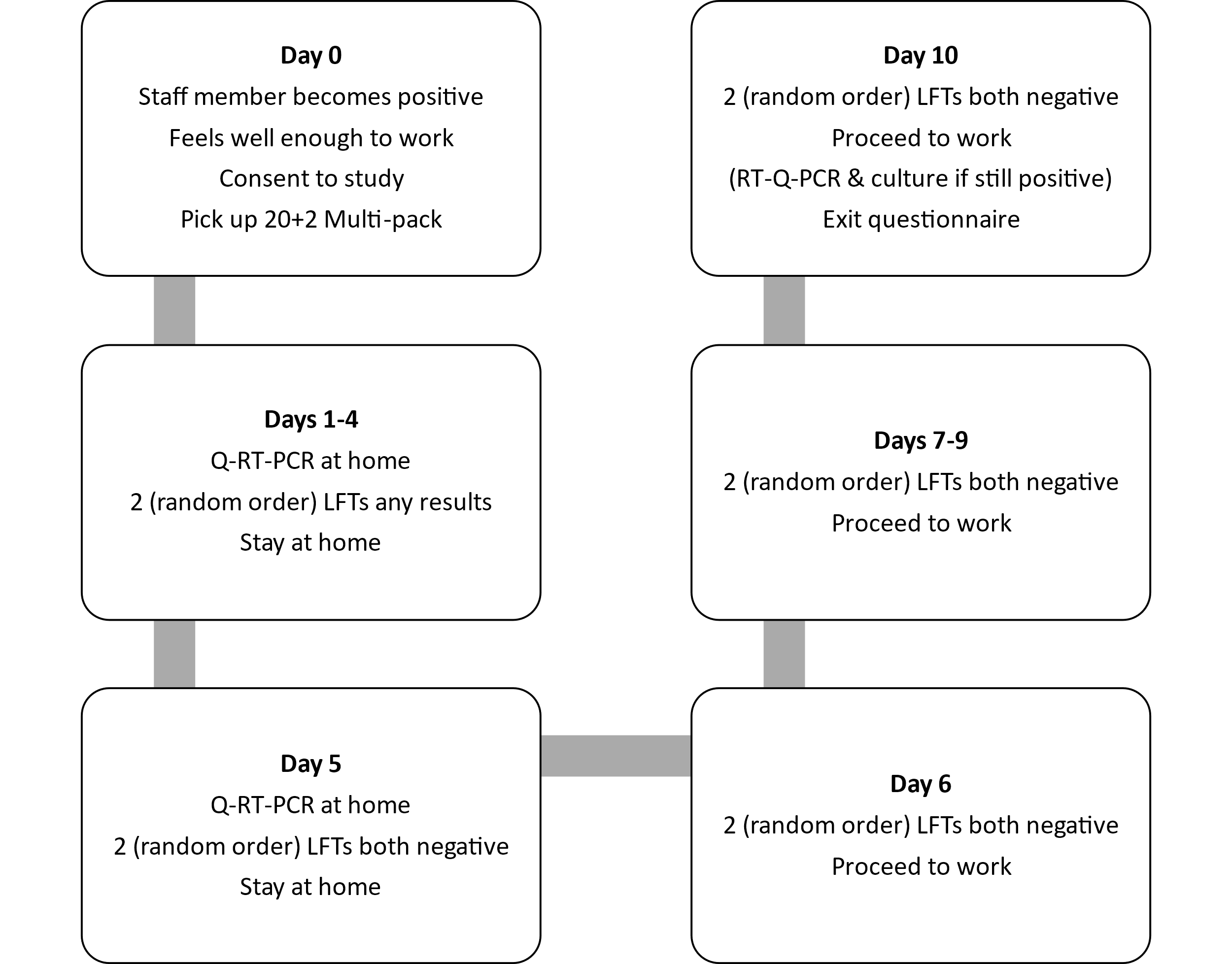


**Figure A1.3. Workflow for participant who became positive at some point during the study or entered the study as a case**

**Appendix 2: Participant information**

A personalised randomisation sheet was inserted in the participants’ pack of swabs/tests with boxes/swabs marked A, B or C, with a typical schedule shown below…

| Day | First test | Second test | Third test |
| --- | --- | --- | --- |
| 1 | Lateral flow "B" | Lateral flow "A" | PCR "C" |
| 2 | Lateral flow "B" | Lateral flow "A" |  |
| 3 | Lateral flow "A" | Lateral flow "B" |  |
| 4 | Lateral flow "A" | Lateral flow "B" |  |
| 5 | Lateral flow "A" | Lateral flow "B" | PCR "C" |
| 6 | Lateral flow "A" | Lateral flow "B" |  |
| 7 | Lateral flow "A" | Lateral flow "B" |  |
| 8 | Lateral flow "A" | Lateral flow "B" |  |
| 9 | Lateral flow "B" | Lateral flow "A" |  |
| 10 | Lateral flow "A" | Lateral flow "B" |  |

The information booklet provided to all participants can be downloaded from: <https://github.com/iain-buchan/cipha/blob/master/SMART_RR_Participant_Information.pdf>

**Appendix 3: Exit Survey**

**On-line form presented to the participant**

SMART Release and Return Questionnaire: Please answer the questions below in relation to your experience of carrying out two rapid tests.

Q1 How easy was the swabbing process?

- Very easy (7)
- Easy (8)
- Neither easy nor difficult (9)
- Difficult (10)
- Very difficult (11)

Q2 How quick was the swabbing process?

- Very quick (7)
- Quick (8)
- Neither quick nor slow (9)
- Slow (10)
- Very slow (11)

Q3 How comfortable was the swabbing process?

- Very comfortable (7)
- Comfortable (8)
- Neither comfortable nor uncomfortable (9)
- Uncomfortable (10)
- Very uncomfortable (11)

Q4 Could you fit taking two rapid tests into your daily routine within an hour of leaving for work?

- Definitely (12)
- Very probably (13)
- Probably (14)
- Probably not (15)
- Definitely not (16)

Q5 How much of a barrier is having to take a throat as well as nose swab for your daily rapid test?

- Not at all (17)
- Slight barrier (18)
- Somewhat of a barrier (19)
- Moderate barrier (20)
- Extreme barrier (21)

Q6 Does using a mobile application to read your lateral flow kit make it easier or harder for you?

- Much easier (17)
- Easier (18)
- Neither easier nor harder (19)
- Harder (20)
- Much harder (21)

Q7 Are there any experiences or suggestions for improvement that you want to share?

________________________________________________________________

Q8 If you were asked to do two tests daily instead of one, would you?

- Yes (16)
- No (17)
- No Preference (18)

Q9 Which mode of testing are you most confident about?

|  | | | 1 - No Confidence | | | | | | 10 - Full Confidence | | | | |
| --- | --- | --- | --- | --- | --- | --- | --- | --- | --- | --- | --- | --- | --- |
|  | 1 | | | 2 | 3 | 4 | 5 | 6 | | 7 | 8 | 9 | 10 |
| Single Test () | | 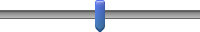 | | | | | | | | | | | |
| Double Test () | | 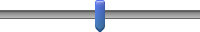 | | | | | | | | | | | |

Below we have added some optional questions about yourself, which would assist us in better understanding your responses. All details covered in this section will be kept separate from the rest of the data, and only be used for internal analysis.

Q10 What sex do you identify most with?

- Non-binary (4)
- Man (5)
- Woman (6)
- Other (7)
- Prefer not to say (8)

Q11 Age:

Q12 Role:

▼ Doctor (1) ... Other (6)

**Results**

311 participants responded to the exit survey between 10^th^ February and 20^th^ July 2022. Their characteristics and responses are given in Table A3. Not all the 311 are reflected in the 226 with testing data to analyse as they may not have returned sufficient data or have been lost to record linkage problems.

**Table A3. Summary of the exit survey**

|  | **Number** | **%** |
| --- | --- | --- |
| **Gender identity** |  |  |
| Male | 77 | 24.7% |
| Female | 229 | 73.7% |
| Preferred not to say | 5 | 1.6% |
| **Role** |  |  |
| Doctors | 57 | 18.5% |
| Nurses | 84 | 27.2% |
| Allied health professionals | 78 | 25.0% |
| Clinical support staff | 25 | 8.2% |
| Administration/clerical staff | 44 | 14.1% |
| Other | 22 | 7.0% |
| **The swabbing process** |  |  |
| Very easy | 154 | 49.6% |
| Easy | 128 | 41.2% |
| Neither easy nor difficult | 26 | 8.4% |
| Difficult | 3 | 0.9% |
| Very difficult | 0 | 0% |
| **Barrier (taking a throat and a nose swab for daily rapid test)** |  |  |
| Not at all | 178 | 57.1% |
| Slight barrier | 80 | 25.6% |
| Somewhat of a barrier | 38 | 12.3% |
| Moderate barrier | 12 | 4.0% |
| Extreme barrier | 3 | 1.0% |
| **Taking two rapid tests into daily routine within an hour of leaving for work** |  |  |
| Definitely | 90 | 28.9% |
| Very probably | 58 | 18.6% |
| Probably | 110 | 35.3% |
| Probably not | 44 | 14.22% |
| Definitely not | 9 | 2.9% |
| **Continue to do two tests daily instead of one** |  |  |
| Yes | 204 | 65.6% |
| No | 57 | 18.2% |
| No preference | 50 | 16.2% |
| **Confidence level for single test (1 = “no confidence” to 10 = “full confidence”)^*^** |  |  |
| Unconfident (<=5) | 25 | 13.4% |
| Confident (>5) | 161 | 86.6% |
| **Confidence level for double test (1 = “no confidence” to 10 = “full confidence”)^*^** |  |  |
| Unconfident (<=5) | 16 | 8.6% |
| Confident (>5) | 169 | 91.4% |
| **Total participants** | **311** | **100%** |

* median scores were 8 for single and 9 for double test: median difference 1 (0.5 to 1; P<0.001).

**Appendix 4: Sample Size Calculation**

Assuming a conservative LFT sensitivity of 0.5 (accepting sensitivity varies as viral load changes over time) for PCR-LFT concordance, the proportion of cases missed on two consecutive days of single lateral flow device testing would then be about 0.25 (0.5^2; with the limitation that within-individual physiological and behavioural factors may break independence). With dual testing this proportion over two days would be about 0.0625 (0.5^4), probably higher due to dependence between two consecutive LFTs done on the same day. We therefore assumed this proportion to be 40% higher (0.0625*1.4=0.09). So, assuming a LFT sensitivity of 0.5, for every 100 PCR positives, 25 cases are expected to be missed by two consecutive single tests versus approximately 9 cases with dual testing (i.e., 4 tests over two consecutive days).

The estimated odds ratio for single versus dual testing is then 3.37 ((0.25/0.75)/(0.09/0.91)) and the proportion of PCR positives with discordant pairs is about 16/100 positive cases (since the 9 negative cases by dual testing would be also negative by single testing).

Assuming these values for the underlying odds ratio and the % of discordant pairs between the two approaches, the number of positive cases required with 80% power at 5% significance to detect a significant difference between the two approaches in the proportion of cases missed is about 164 positive cases.[A4i] We raised it to 200 to account for loss to follow-up. Using only a contact cohort with about 10% case rate this would require a sample of 2000. We eventually recruited 1929 participants.

Table A4.1 shows the sample size for various values of sensitivity and for different scenarios regarding the dependence between two consecutive LFTs done on the same day. Note that the sample size required varies substantially with the within-individual correlation, which is unknown. We assumed some level of dependence between two tests conducted between minutes of each other, but not tests conducted on consecutive days, and had ignored the manufacturer factor.

Table A4.1 assumes that the two LFTs achieve the same level of sensitivity. Deviation from this assumption (i.e., allowing some degree of differentiation in sensitivities) is not expected to alter the sample size reported in Table A4.1 significantly. The power that can be achieved to detect a 15% drop in sensitivity over a two-day period with nose-only swabbing, when compared to nose-throat swabbing with a kit from a different manufacturer but with equivalent device sensitivity, is provided in Table A4.2 for different sample sizes and values of sensitivity. Subsequent reports of Innova and Orent Gene LFT sensitivity relevant to PCR showed similar profiles across different levels of viral load and different variants, but with Orient Gene the more sensitive.[A4ii-iii] Both tables use equation (7.1) in Machin et al. (2011)[A4i] based on discordance between results.

[A4i] Machin D, Campbell MJ, Tan SB, Tan SH. *Sample Size Tables for Clinical Studies*. John Wiley & Sons; 2011.

[A4ii] Eyre DW, Futschik M, Tunkel S, Wei J, Cole-Hamilton J, Saquib R, et al. Performance of antigen lateral flow devices in the UK during the alpha, delta, and omicron waves of the SARS-CoV-2 pandemic: a diagnostic and observational study. *The Lancet Infectious Diseases* 2023;23(8):922–32. Doi: 10.1016/S1473-3099(23)00129-9.

[A4iii] Performance of lateral flow devices during the COVID-19 pandemic. GOV.UK. Available at https://www.gov.uk/government/publications/lateral-flow-device-performance-data/performance-of-lateral-flow-devices-during-the-covid-19-pandemic. Accessed May 3, 2023.

**Table A4.1. PCR positive sample size versus LFT sensitivity to detect a significant difference between dual and single LFT testing in the proportion of discordant cases with 80% power and 5% significance level.**

| LFT Sensitivity | Ps$\equiv$P_single=Prob (negative LFT based on a single test \| positive PCR) | Ps$\equiv$P_dual=Prob (dual negative LFTs based on 2 consecutive tests within minutes \| positive PCR) | P1=Prob (negative LFTs on 2 consecutive days \| positive PCR)=Ps^2^ | P2=Prob (dual negative LFTs on 2 consecutive days \| positive PCR)=Pd^2^ | Odds Ratio | Proportion of discordant pairs | Number of PCR positives required |
| --- | --- | --- | --- | --- | --- | --- | --- |
| Assuming independence (P_dual=P_single*P_single) | | | | | | | |
| 0.7 | 0.3 | 0.09 | 0.09 | 0.0081 | 12.1 | 8.2% | 131 |
| 0.6 | 0.4 | 0.16 | 0.16 | 0.0256 | 7.25 | 13.4% | 99 |
| 0.5 | 0.5 | 0.25 | 0.25 | 0.0625 | 5.0 | 18.8% | 92 |
| 0.4 | 0.6 | 0.36 | 0.36 | 0.1296 | 3.78 | 23.0% | 98 |
| Assuming some level of dependence between two tests conducted within minutes | | | | | | | |
| P_dual=P_single*P_single*1.2 | | | | | | | |
| 0.7 | 0.3 | 0.11 | 0.09 | 0.0117 | 8.07 | 7.8% | 159 |
| 0.6 | 0.4 | 0.192 | 0.16 | 0.0369 | 4.98 | 12.3% | 142 |
| 0.5 | 0.5 | 0.3 | 0.25 | 0.09 | 3.37 | 16.0% | 164 |
| 0.4 | 0.6 | 0.432 | 0.36 | 0.1866 | 2.45 | 17.3% | 254 |
| Pdual=Psingle*Psingle*1.4 | | | | | | | |
| 0.7 | 0.3 | 0.126 | 0.09 | 0.0159 | 6.13 | 7.4% | 202 |
| 0.6 | 0.4 | 0.192 | 0.16 | 0.0502 | 3.61 | 11.0% | 221 |
| 0.5 | 0.5 | 0.35 | 0.25 | 0.1225 | 2.39 | 12.75% | 365 |
| 0.4 | 0.6 | 0.504 | 0.36 | 0.2540 | 1.65 | 10.1% | 1223 |

**Table A4.2. Power to detect a 15% drop in sensitivity (or higher drop) over a two-day period with a nose-only swabbing LFT brand (when compared to a nose-throat swabbing LFT from a different manufacturer with equivalent device sensitivity) with 5% significance level.**

| LFT sensitivity (nose-throat) | LFT sensitivity (nose-throat) over two days | LFT sensitivity (nose-only) over two days | Number of positive PCR cases | Power |
| --- | --- | --- | --- | --- |
| 0.7 | 0.91 | 0.77 (=0.91*0.85) | 200 | 96% |
| 0.6 | 0.84 | 0.714 | 200 | 85% |
| 0.5 | 0.75 | 0.638 | 200 | 68% |
| 0.4 | 0.64 | 0.555 | 200 | 50% |
| 0.7 | 0.91 | 0.77 | 250 | 98% |
| 0.6 | 0.84 | 0.714 | 250 | 92% |
| 0.5 | 0.75 | 0.638 | 250 | 77% |
| 0.4 | 0.64 | 0.555 | 250 | 58% |
| 0.7 | 0.91 | 0.77 | 300 | 99% |
| 0.6 | 0.84 | 0.714 | 300 | 96% |
| 0.5 | 0.75 | 0.638 | 300 | 85% |
| 0.4 | 0.64 | 0.555 | 300 | 66% |

**Appendix 5: Viral Culture and Bioinformatics Analysis**

Swabs were collected by the study team and transported directly to a University of Liverpool Containment Level 3 (CL3) facility. The universal transport media (UTM) was split into cryovials with 250 μL aliquots. 1 aliquot used for viral culture and the remainder frozen at -80°C. Calu3 cells, cultured at 10^5 cells/well in 24 well plates, were inoculated for viral culture with 100μl UTM after filtration using a 0.2 μm filter and centrifugation at 12000 x g for 4mins to remove bacterial contaminants. The filtered sample was diluted 1:1 with Dulbecco's Modified Eagle Medium (DMEM) +2% Fetal Bovine Serum (FBS) + Plasmocin (clarithromycin)/Gentamicin/Amphotericin B. A mock control of medium only was on each plate. The remaining filtered sample aliquot was frozen down.

After incubation for 30 minutes at 37°C, in 5% CO_2_, 500μL DMEM/2% FBS with Plasmocin/Gentamicin/Amphotericin was added to each well and plates returned to the incubator at 37°C, 5% CO_2_ for 3 days.

After 3 days plates were checked for cytopathic effect (CPE). If CPE was apparent 250 μL of supernatant was removed from the plate and added to 750μLTrizol-LS for RNA extraction. Any remaining supernatant was stored at -80°C. If no CPE was visible after 3 days, 250 μL of the supernatant was taken from the well and added to a fresh well of Calu3 cells for a 2^nd^ passage. The plate was placed back into the incubator at 37°C, 5% Co2 for a further 3 days. If after 3 days there was still no CPE visible, 250 μL was taken from each well and added to 750 μL Trizol-LS for RNA extraction. Any remaining supernatant was stored at -80°C.

For RNA Extraction, Thermofisher’s Phasemaker™ tubes and TRizol reagent was used, following the manufacturer’s instructions. After precipitation and drying, the RNA was finally resuspended in 50 μL of RNase-free water and incubated in a heat block at 60°C for 15 minutes. RNA was used for amplicon sequencing by MinION, on an Oxford Nanopore GridION device using the ARTIC V4.1 primer scheme and ligation kits (SQK-LSK109).[A5i]

The workflow is summarised in Figure A5.1.


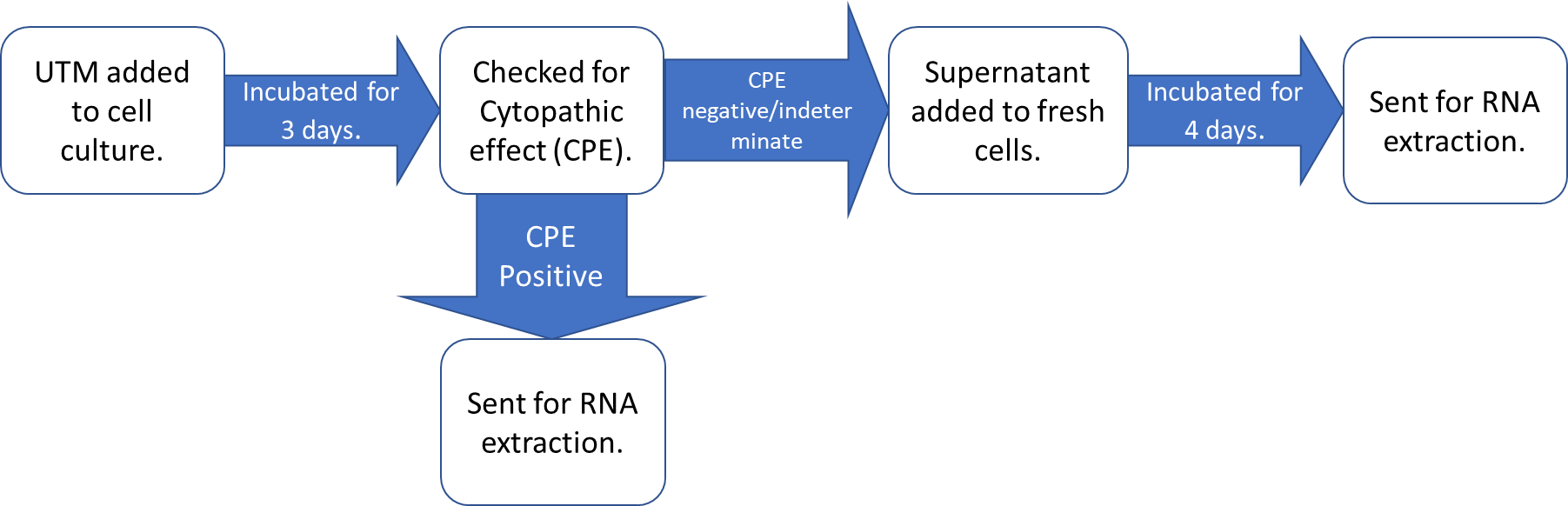


**Figure A5.1: Viral culture laboratory process**

A total of 41 Patients had swabs taken for laboratory analysis. 31 came from patients still testing LFT positive at day 5-7. 10 came from patients testing LFT negative at day 5-7.

Cultures were considered positive if CPE was present. Because CPE is not always present in viral cultures, especially with the omicron variant, culture supernatants had RNA isolated and sequenced culture supernatants had RNA isolated and sequenced by MinION. If cultures were indeterminate for CPE, or if N gene sub genomic RNA (sgRNA) was identified using a published method.[A5ii] In one case, both indeterminate CPE and N sgRNA were identified. This sample had multiple other sgRNAs detected and was considered positive. The remainder of the samples had either indeterminate CPE or N sgRNA but not both, lower levels of N sgRNA, and fewer sgRNAs detected. In these cases we were unable to prove the presence of SARS-CoV-2, these have therefore been considered indeterminate by either CPE or N sgRNA. Table A5 shows a summary of these results.

| **Culture result** | **LFT result** | **Laboratory result** | **N** |
| --- | --- | --- | --- |
| Positive | Positive | CPE positive | 5 |
|  |  | CPE indeterminate, N sgRNA positive, 4 sgRNAs | 1 |
| Indeterminate | Positive | Indeterminate CPE | 9 |
|  |  | N sgRNA positive (low level, ≤ 3 sgRNAs) | 2 |
|  | Negative | Indeterminate CPE | 2 |
|  |  | N sgRNA positive (low level, ≤ 3 sgRNAs) | 2 |
| Negative | Positive | All negative | 14 |
|  | Negative | All negative | 6 |

**Table A5.1: Classification of culture result by LFT status. CPE = cytopathic effect.**

**Bioinformatics Analysis**

Fastq reads were analysed using the ARTIC[A5i] bioinformatic pipeline and lineages were called with Pangolin.[A5iii] LeTRS[A5ii] was used to assess the presence of sgmRNA, indicative of active viral transcription. Sequencing reads were mapped onto a known SARS-CoV-2 genome (GenBank sequence accession: NC_045512.2) using Minimap2 v 2.24-r1122 with parameters “-a -x map-ont".[A5iv] The sorted output was used to count the number of reads mapped on the virus genome using SAMtools v1.9 with “flagstat” option.[A5v] The “genomecov” function in bedtools v2.29.2 was used to calculate the coverage of reads mapped on the virus.[A5vi] LeTRS v2.2.1 was applied to analyse sub-genomes in the sequenced samples with the options set to “-pool 0 -Rtch cDNA -mode nanopore”.[A5ii]

[A5i] SARS-CoV-2 V4.1 update for Omicron variant - Laboratory. ARTIC Real-Time Genomic Surveillance. Available at https://community.artic.network/t/sars-cov-2-v4-1-update-for-omicron-variant/342. Accessed October 10, 2023, 2021.

[A5ii] Dong X, Penrice-Randal R, Goldswain H, Prince T, Randle N, Donovan-Banfield I, et al. Analysis of SARS-CoV-2 known and novel sub-genomic mRNAs in cell culture, animal model, and clinical samples using LeTRS, a bioinformatic tool to identify unique sequence identifiers. *GigaScience* 2022;11:giac045. Doi: 10.1093/gigascience/giac045.

[A5iii] O’Toole Á, Pybus OG, Abram ME, Kelly EJ, Rambaut A. Pango lineage designation and assignment using SARS-CoV-2 spike gene nucleotide sequences. *BMC Genomics* 2022;23(1):121. Doi: 10.1186/s12864-022-08358-2.

[A5iv] Li H. Minimap2: pairwise alignment for nucleotide sequences. *Bioinformatics* 2018;34(18):3094–100. Doi: 10.1093/bioinformatics/bty191.

[A5v] Li H, Handsaker B, Wysoker A, Fennell T, Ruan J, Homer N, et al. The Sequence Alignment/Map format and SAMtools. *Bioinformatics* 2009;25(16):2078–9. Doi: 10.1093/bioinformatics/btp352.

[A5vi] Quinlan AR, Hall IM. BEDTools: a flexible suite of utilities for comparing genomic features. *Bioinformatics* 2010;26(6):841–2. Doi: 10.1093/bioinformatics/btq033.

**Appendix 6: Additional Data and Classifications**

The number of days each participant contributed dual LFT results is shown in Table A6.1. Some people did not contribute many days of dual LFT results while others stopped recording test results once they turned from positive to negative, contributing to the low proportion of participants who provided dual tests on all 10 days (20.4%). This sparsity does not affect the main inference of the study which uses test result pairs and not individuals (or their time courses) as the units of analysis.

**Table A6.1. Numbers of individuals reporting dual LFT results by study day**

| **Days with dual LFT results recorded** | **Number of individuals (%)** |
| --- | --- |
| 1 | 23 (10.2%) |
| 2 | 10 (4.4%) |
| 3 | 16 (7.1%) |
| 4 | 15 (6.6%) |
| 5 | 21 (9.3%) |
| 6 | 15 (6.6%) |
| 7 | 13 (5.8%) |
| 8 | 28 (12.4%) |
| 9 | 39 (17.3%) |
| 10 | 46 (20.4%) |
| **Total** | **226 (100%)** |

167 participants were regarded as positive cases at any time during the study. They were defined as cases at baseline if they had a positive PCR from attendance for swabbing at the staff testing centre (when they consented to participate in the study) or reported dual LFT positive results on day 1. They were defined as subsequent positive cases if they reported dual LFT positive results on any of days 2-10. An additional PCR on day 5 was sought from participants via postal return, however, this was not part of the main analysis. PCR results had only the date of processing and time periods between swabbing and processing may have varied, therefore we classified PCR results as swabbed on day 1 if they were processed on days 1-3 and swabbed on day 5 if they were processed on days 5-7 from the date of consent. PCR results were not available for all participants. Table A6.2 gives a breakdown of LFT results by case status at baseline by PCR result or by dual lateral flow test result where the PCR result is not available.

**Table A6.2. Breakdown of participants’ case status by PCR and LFT results**

| **PCR Result at Baseline** | **n (%)** | **Baseline status following logic rules** | **n (%)** | **First day with at least one positive LFT result for those classed as positive at any point during the study** | **n (%)** | **PCR Result on day 5** | **n (%)** |
| --- | --- | --- | --- | --- | --- | --- | --- |
| Positive | 125 (55.3%) | n/a | | 1 | 85 (68.0%) | Positive | 70 (56.0%) |
|  |  |  |  | 2 | 25 (20.0%) | Negative | 3 (2.4%) |
|  |  |  |  | 3 | 9 (7.2%) | Equivocal | 1 (0.8%) |
|  |  |  |  | 4 | 2 (1.6%) | No result recorded | 51 (40.8%) |
|  |  |  |  | 5 | 1 (0.8%) |  | |
|  |  |  |  | 6 | 1 (0.8%) |  |  |
|  |  |  |  | 7 | 0 (0%) |  |  |
|  |  |  |  | 8 | 1 (0.8%) |  |  |
|  |  |  |  | 9 | 0 (0%) |  |  |
|  |  |  |  | 10 | 0 (0%) |  |  |
|  |  |  |  | None | 1 (0.8%) |  |  |
| Negative | 65 (28.8%) | n/a | | 1 | 2 (3.1%) | Positive | 11 (16.9%) |
|  |  |  |  | 2 | 5 (7.7%) | Negative | 25 (38.5%) |
|  |  |  |  | 3 | 2 (3.1%) | Equivocal | 2 (3.1%) |
|  |  |  |  | 4 | 1 (1.5%) | No result recorded | 27 (41.5%) |
|  |  |  |  | 5 | 4 (6.2%) |  | |
|  |  |  |  | 6 | 0 (0%) |  |  |
|  |  |  |  | 7 | 1 (1.5%) |  |  |
|  |  |  |  | 8 | 1 (1.5%) |  |  |
|  |  |  |  | 9 | 0 (0%) |  |  |
|  |  |  |  | 10 | 0 (0%) |  |  |
|  |  |  |  | None | 49 (75.4%) |  |  |
| Unknown (equivocal or no record) | 36 (15.9%) | Positive* | 12 (5.3%) | 1 | 12 (33.3%) | Positive | 13 (36.1%) |
|  |  | Negative* | 9 (4.0%) | 2 | 9 (25.0%) | Negative | 2 (5.6%) |
|  |  | Unknown** | 15 (6.6%) | 3 | 2 (5.6%) | Equivocal | 1 (2.8%) |
|  |  |  | | 4 | 1 (2.8%) | No result recorded | 20 (55.6%) |
|  |  |  |  | 5 | 1 (2.8%) |  | |
|  |  |  |  | 6 | 1 (2.8%) |  |  |
|  |  |  |  | 7 | 0 (0%) |  |  |
|  |  |  |  | 8 | 0 (0%) |  |  |
|  |  |  |  | 9 | 0 (0%) |  |  |
|  |  |  |  | 10 | 0 (0%) |  |  |
|  |  |  |  | None | 10 (27.8%) |  |  |
| **Total** | **226 (100)** |  | | | | | |

*Based on dual LFT results on day 1. **Either discordant LFT results, single or no LFT results on day 1.

**Table A6.3. Numbers (%) of PCR results linked to study days 1 and 5.**

| **PCR Results** | **Day 1** | **Day 5** |
| --- | --- | --- |
| Positive | 109  (48.2%) | 94  (41.6%) |
| Negative | 42  (18.6%) | 30  (13.3%) |
| Equivocal | 0  (0%) | 4  (1.8%) |
| No Result | 75  (33.2%) | 98  (43.4%) |
| **Total** | **226**  **(100%)** | **226**  **(100%)** |

There were 10 (4.4%) participants who tested negative with dual LFTs on day 5 and had a positive day 5 PCR result (Table A6.4).

**Table A6.4. Numbers (5) of LFT by PCR results on day 5.**

|  | | **Day 5 PCR result** | | | | |
| --- | --- | --- | --- | --- | --- | --- |
|  |  | **Positive** | **Negative** | **Equivocal** | **No result** | **Total** |
| **Day 5 LFT result** | **Positive*** | 73  (32.3%) | 1  (0.4%) | 0  (0%) | 30  (13.3%) | 104  (46.0%) |
|  | **Negative** | 10  (4.4%) | 22  (9.7%) | 3  (1.3%) | 20  (8.8%) | 55  (24.3%) |
|  | **Unknown**** | 11  (4.9%) | 7  (3.1%) | 1  (0.4%) | 48  (21.2%) | 67  (29.6%) |
|  | **Total** | 94  (41.6%) | 30  (13.3%) | 4  (1.8%) | 98  (43.4%) | 226  (100%) |

*Either one or both LFT results were positive. ** Includes dual tests which include an equivocal result as well as where no dual LFT results were recorded.

Table A6.5 shows the frequency of positivity for participants for days 5 to 10 from the first recorded positive test. Note that these results are right censored for those who became positive during the study as the follow-up period was shorter than for someone who was positive at study entry.

**Table A6.5. Frequency of positivity on days 5-10 from first recorded positive test.**

| **Time** | **Status** | **n (%)** |
| --- | --- | --- |
| Anytime | Case within study | 167  (100%) |
| Day 5 | Positive | 110  (65.9%) |
|  | No further positive tests^*^ | 57  (34.1%) |
| Day 6 | Positive | 93  (55.7%) |
|  | No further positive tests^*^ | 74  (44.3%) |
| Day 7 | Positive | 75  (44.9%) |
|  | No further positive tests^*^ | 92  (55.1%) |
| Day 8 | Positive | 49  (29.3%) |
|  | No further positive tests^*^ | 118  (70.7%) |
| Day 9 | Positive | 32  (19.2%) |
|  | No further positive tests^*^ | 135  (80.8%) |
| Day 10 | Positive | 15  (9.0%) |
|  | No further positive tests^*^ | 152  (91.0%) |

*Includes individuals with dual negative LFT results and individuals with no further tests recorded.

Table A6.6 shows there were very few discordant LFT result pairs observed among those who never tested positive.

**Table A6.6. LFT results by brand and days from consent for those participants who never became positive.**

|  | **Day from consent** | | | | | | | | | |
| --- | --- | --- | --- | --- | --- | --- | --- | --- | --- | --- |
| **Dual LFT results** | **1** | **2** | **3** | **4** | **5** | **6** | **7** | **8** | **9** | **10** |
| **Concordant** | 38  (100%) | 43  (97.7%) | 38  (95.0%) | 32  (97.0%) | 36  (100%) | 30  (100%) | 27  (100%) | 24  (100%) | 22  (100%) | 21  (100%) |
| ***Both positive*** | *0*  *(0%)* | *0*  *(0%)* | *0*  *(0%)* | *0*  *(0%)* | *0*  *(0%)* | *0*  *(0%)* | *0*  *(0%)* | *0*  *(0%)* | *0*  *(0%)* | *0*  *(0%)* |
| ***Both negative*** | *38*  *(100%)* | *43*  *(97.7%)* | *38*  *(95.0%)* | *32*  *(97.0%)* | *36*  *(100%)* | *30*  *(100%)* | *27*  *(100%)* | *24*  *(100%)* | *22*  *(100%)* | *21*  *(100%)* |
| **Discordant** | 0  (0%) | 1  (2.3%) | 2  (5.0%) | 1  (3.0%) | 0  (0%) | 0  (0%) | 0  (0%) | 0  (0%) | 0  (0%) | 0  (0%) |
| ***Orient Gene positive*** | *0*  *(0%)* | *0*  *(0%)* | *0*  *(0%)* | *0*  *(0%)* | *0*  *(0%)* | *0*  *(0%)* | *0*  *(0%)* | *0*  *(0%)* | *0*  *(0%)* | *0*  *(0%)* |
| ***Innova positive*** | *0*  *(0%)* | *1*  *(2.3%)* | *2*  *(5.0%)* | *1*  *(3.0%)* | *0*  *(0%)* | *0*  *(0%)* | *0*  *(0%)* | *0*  *(0%)* | *0*  *(0%)* | *0*  *(0%)* |
| **Total** | 38 | 44 | 40 | 33 | 36 | 30 | 27 | 24 | 22 | 21 |
